# Validation of a wireless patch sensor to monitor mobility tested in both an experimental and a hospital setup: A cross-sectional study

**DOI:** 10.1371/journal.pone.0206304

**Published:** 2018-10-25

**Authors:** Niek Koenders, Joost P. H. Seeger, Teun van der Giessen, Ties J. van den Hurk, Indy G. M. Smits, Anne M. Tankink, Maria W. G. Nijhuis - van der Sanden, Thomas J. Hoogeboom

**Affiliations:** 1 Department of Physiotherapy, Radboud Institute for Health Sciences, Radboud university medical center, Nijmegen, Gelderland, the Netherlands; 2 Research group Musculoskeletal Rehabilitation, HAN university of applied sciences, Nijmegen, Gelderland, the Netherlands; 3 IQ healthcare, Radboud Institute for Health Sciences, Radboud university medical center, Nijmegen, Gelderland, the Netherlands; Federal University of Pelotas, BRAZIL

## Abstract

**Purpose:**

To assess the concurrent validity of a wireless patch sensor to monitor time lying, sitting/standing, and walking in an experimental and a hospital setup.

**Methods:**

Healthy adults participated in two testing sessions: an experimental and real-world hospital setup. Data on time lying, sitting/standing, and walking was collected with the HealthPatch and concurrent video recordings. Validity was assessed in three ways: 1. test for mean differences between HealthPatch data and reference values; 2. Intraclass Correlation Coefficient analysis (ICC 3.1 agreement); and 3. test for mean differences between posture detection accuracies.

**Results:**

Thirty-one males were included. Significant mean differences were found between HealthPatch data and reference values for sitting/standing (mean 14.4 minutes, reference: 12.0 minutes, p<0.01) and walking (mean 6.4 minutes, reference: 9.0 minutes, p<0.01) in the experimental setup. Good correlations were found between the HealthPatch data and video data for lying (ICC: 0.824) and sitting/standing (ICC: 0.715) in the hospital setup. Posture detection accuracies of the HealthPatch were significantly higher for lying and sitting/standing in the experimental setup.

**Conclusions:**

Overall, the results show a good validity of the HealthPatch to monitor lying and poor validity to monitor sitting/standing or walking. In addition, the validity outcomes were less favourable in the hospital setup.

## Introduction

Low mobility during hospital stay is common in patients [1] and independently related to poor functional outcomes such as reduced pulmonary function, decreased strength, functional decline, and increased risk on disability in activities of daily living [2–6]. Interventions aiming to decrease the risk on disability and functional decline show good results by increasing the amount of in-hospital mobility [7–10]. Mobility of patients during hospital stay is assessed with a wide variety of methods such as accelerometry [2, 11], structured observations using behavioural mapping protocols [6, 12, 13], and interviews [3]. Accelerometry has a great potential to determine the amount of mobility in patients during hospital stay due to its ability to provide easy and continuously data collection in both brief and lengthy periods of mobility. However, the validity of such accelerometry in the hospital setup is largely unknown [1, 14].

**Fig 1:**
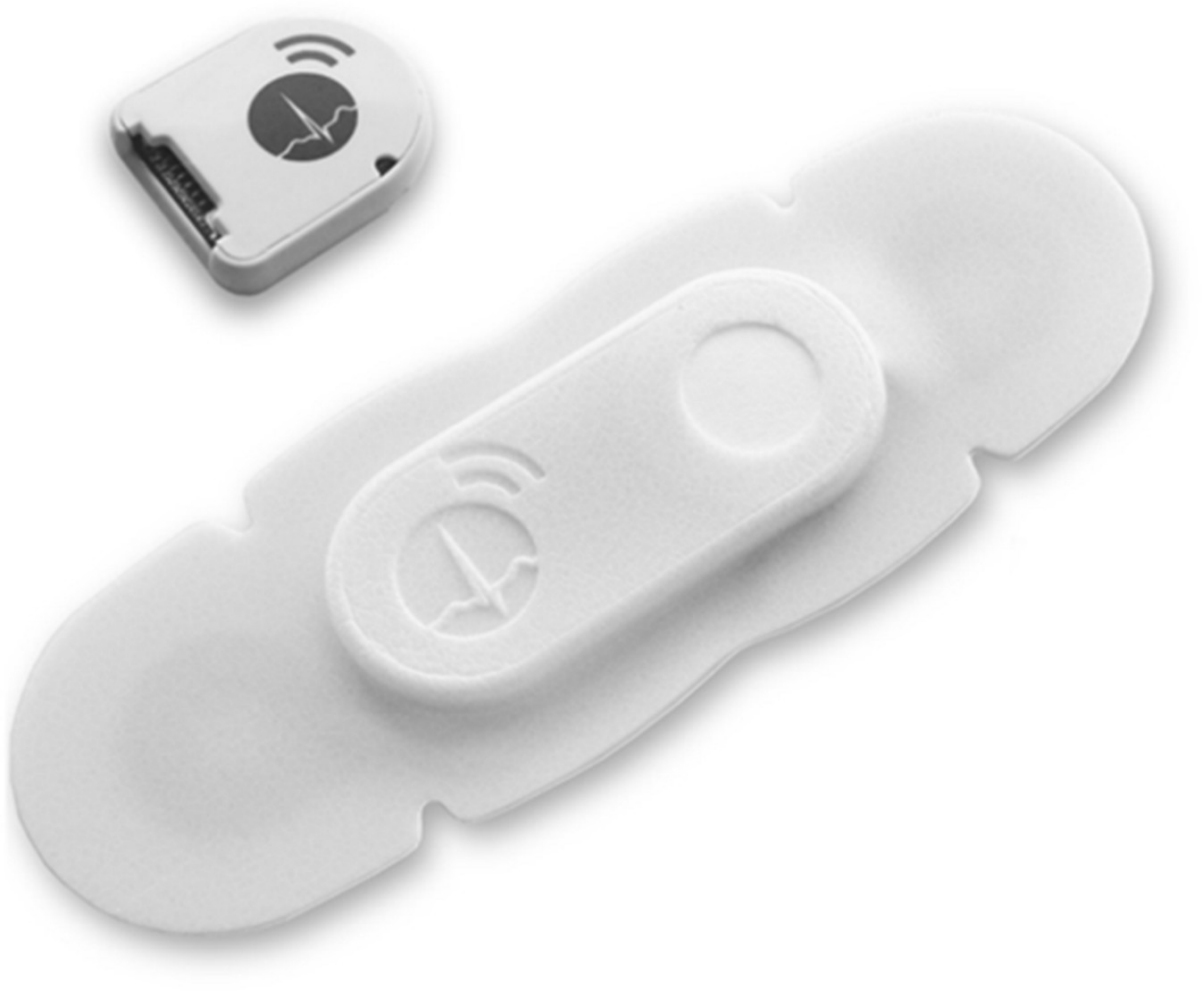
The reusable wireless HealthPatch sensor (left) and disposable HealthPatch (right) [15].

The HealthPatch [15] (Fig 1) is a wireless patch sensor with an accelerometer and equipped for use in hospitals. The HealthPatch is developed to allow thorough evaluation of inpatient mobility (i.e. lying, sitting/standing, and walking) while providing additional data on vital parameters such as heart rate, heart rate variability and respiratory rate. The study of Chan et al. [15] shows promising results with high posture detection accuracies of the HealthPatch in an experimental setup. However, the validity of a measurement device is depending on the context in which it is established [16]. The use of accelerometry in an experimental setup, characterized by scripted activities in a specific order, might misrepresent validity outcomes when compared to real-world conditions [17]. The application of accelerometry in a hospital setup might be considered as a new situation as the context evokes other movement patterns (i.e. lying on the side or sitting on the edge of a hospital bed) [14] and short periods of movement [11, 18]. Therefore, the HealthPatch should specifically be assessed for validity purposes in a setup as close to a real-world hospital setup as possible [19–21]. As the research group considered video recordings in patients during hospitalization too intrusive in this phase of validation, the aim of the current study is to assess the concurrent validity of the HealthPatch to monitor the time lying, sitting/standing, and walking tested in an experimental and real-world hospital setup in healthy adults.

## Materials and methods

### Study design

A cross-sectional, experimental study design was used to assess the concurrent validity of the HealthPatch (Vital Connect, CA, United States of America) (Fig 1). All participants participated in two conditions: firstly a real-world hospital setup, secondly an experimental setup. Validity is defined by the Consensus-based Standards for the selection of health Measurement Instruments (COSMIN) group as ‘the degree to which an instrument truly measures the construct it purports to measure’ [16]. The domain of validity is operationalized in three sub-domains, in which concurrent validity is defined as ‘the degree to which the scores of a measurement instrument are an adequate reflection of a golden standard’ [16]. The current study used video data as the golden standard for assessment of mobility in both an experimental and a hospital setup.

### Participants’ characteristics and recruitment

A convenience sample of students was recruited between February 2017 and July 2017 at the HAN university of applied sciences, Nijmegen, the Netherlands. Students were informed and asked to participate via social media, e-mail, and their digital learning environment. Healthy male students between 18 and 30 years were eligible for inclusion. Healthy was operationalized as the absence of cognitive or physical disability to lie on a bed, sit, stand, and walk for a total of sixty minutes. The current study aimed to include at least 30 participants, which is considered fair in the COSMIN checklist [19]. Principles of the Declaration of Helsinki (64^th^ version, 19-10-2013) were followed and ethical approval was granted by the advisory board Practice oriented research, Faculty of Health, Behaviour and Society, HAN university of applied sciences, Nijmegen, the Netherlands (EACO 57.02/17). Written informed consent for study inclusion and anonymous collection of data was obtained from each respondent.

### Measurement procedure

The measurement procedure started with a standard measurement of body weight (Seca 761), length, and breast girth. Horizontal placement of the patch on the skin at left midclavicular line under the pectoralis minor muscle (position 3) showed the highest accuracy for posture detection in previous research [15] and was for that reason used in all participants. Thereafter, each participant participated for thirty minutes in both the experimental and the hospital setup, resulting in sixty minutes of HealthPatch and video data per participant. Mobility was videotaped with two cameras (GoPro HERO 4) in two different angles to ensure full data capture.

In the experimental setup, participants were instructed by the observers (TG, TvdH, IS, AT) to change level of mobility after standardized blocks of three minutes without breaks according to a predefined format: lying on the hospital bed (block 1, 7, 8), sitting on the hospital bed (block 2, 6, 9), walking in the hospital room (block 3, 5, 10); sitting on the comfortable chair (block 4). In the real-world hospital setup, the observers provided no specific instructions to change the mobility of participants. In addition, participants were not informed about the purpose of measurements to empower natural behaviour. The hospital room had a surface of 30m^2^, with a hospital bed, nightstand, table, two seats, and a closet (S1 Fig). The participants were able to listen to music and watch television from the bed, which is identical to a real-world hospital setup. Drinks, journals and books were provided at the table.

### Outcomes

HealthPatch data, device under investigation–The primary outcome in the current study was time lying, sitting/standing, or walking. The HealthPatch continuously measured level of mobility with a 3-axis micro electro-mechanical system accelerometer resulting in counts with a frequency of 1 Hertz [15]. The time lying, sitting/standing, or walking was saved as one count per second. The data was collected and transmitted instantly by the wireless HealthPatch sensor to an IPad by Bluetooth and securely stored in private cloud data files.

Video data, golden standard–Analysis of video recordings on mobility in participants was considered as the golden standard to determine actual time lying, sitting/standing, or walking of the participants. Second-by-second analysis of the video data was performed by the observers (TG, TvdH, IS, AT) and reported in a standardized case report form. In this form, the observers reported the time lying, sitting/standing, and walking of the participants. In addition, details were provided on the start and end of measurements, problems with data collection, and name of observers. Definitions of different levels of mobility were described by Pedersen et al. [11] and used in the current study to improve interrater assessment of video data. At last, the four observers assessed 270 minutes of new videotaped pilot test data in which the inter-observer reliability was calculated.

### Data analysis

HealthPatch data was stored (AT, TvdH) and double checked (AT, TvdH, NK) for incorrect data and missing values. In case of incorrect or missing data, data files (HealthPatch data) and case report forms (video data) were checked to correct the data entry. Prior to data analysis, a syntax was written (AT, TH, NK) to provide independent data analysis in IBM SPSS statistics, version 23 [22]. Descriptive statistics (mean, range) were used to describe the participant characteristics. All time in different levels of mobility was expressed in minutes.

#### Experimental setup

HealthPatch data on individual level were presented and analyzed with a scatter plot to observe differences in HealthPatch data between participants. Mean differences were calculated between the HealthPatch data and reference values (observed time lying, sitting/standing, walking) with a one sample t-test. The video data showed exactly three minutes lying, sitting/standing or walking per block in all participants as predetermined, securing solid use of the reference values. Outcomes with a two-tailed p-value < 0.05 were considered significant.

#### Hospital setup

The HealthPatch data were analyzed for correlation with the golden standard video data in the hospital setup. Firstly, data on individual level were presented for lying, sitting/standing and walking with a scatter plot of both HealthPatch and video data. Secondly, Intraclass Correlation Coefficients (ICCs) were calculated between the HealthPatch and video data using a two-way mixed effect model (ICC 3.1 agreement) with 95% confidence intervals (CI). A correlation of ICC < 0.70 was considered poor or moderate, ICC ≥ 0.70 as good and ICC ≥ 0.90 as excellent [23, 24]. Thirdly, measurement error was investigated using Bland Altman 95% limits of agreements (LOA) and visual inspection of measurement error patterns [24]. The limits of agreement illustrate the range and magnitude of the differences between HealthPatch outcomes and video observations [25].

#### Comparison experimental and hospital setup

A comparison of ICC outcomes between the experimental and hospital setup would provide information on differences in outcomes related to the specific setup. However, correlation coefficients cannot be calculated for data in the experimental setup as there was no variance in time lying, sitting/standing, and walking in all participants. With no variance in the independent variable, the standardized time lying, sitting/standing, or walking, a correlation coefficient cannot be calculated [24]. An independent samples t-test was found to adequately provide information on the (significant) differences between outcomes of the experimental and hospital setup. Therefore, posture detection accuracies were computed on data collected in both the experimental and the hospital setup. The accuracies were calculated as the percent agreement between the HealthPatch and video data.

## Results

Thirty-one males were included in the current study with a mean age of 22 years, ranging between 18 and 29 years. The demographics were: mean weight 80 kilograms (range: 60-104kg), mean length 183 centimetres (range: 171-195cm), and mean breast girth 90 centimetres (range: 81-100cm). HealthPatch data were missing on four participants in the experimental setup (participant 9, 17, 19, 24) and hospital setup (participant 6, 7, 24, 25) as a result of connectivity problems. One HealthPatch (case 24) disconnected as the chest hair of the participant impaired the connection between the HealthPatch and his skin. Three participants sat with their arms folded for their chest, causing the HealthPatch to stop register and transfer data. No explanation was found for the missing data in the remaining four participants. As a result, the current study included data on 27 participants (810 minutes) per setup. Analysis of the inter-observer reliability on pilot test video data showed ICC’s (agreement) between 0.971 and 0.999.

### Concurrent validity–experimental setup

**Fig 2:**
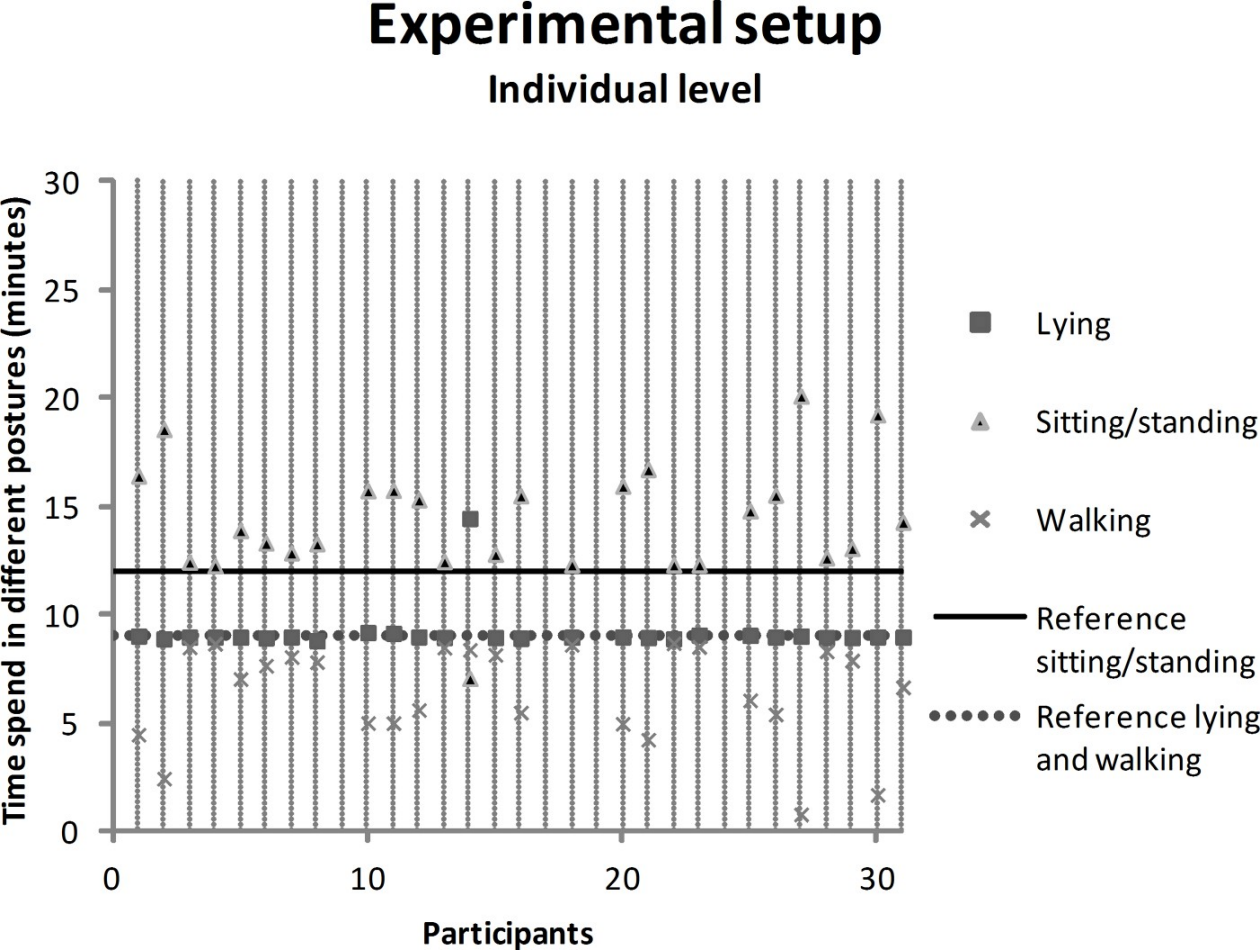
Scatter plot showing the individual HealthPatch data in the experimental setup, with a reference line for the actual time that participants were lying (9 minutes, dashed line), sitting/standing (12 minutes, solid line), or walking (9 minutes, dashed line).

Fig 2 shows the HealthPatch data of the participants in the experimental setup on individual level. The minimum and maximum recorded time lying at individual level were respectively 8.8 and 14.5 minutes, sitting/standing 7.1 and 20.1 minutes, and walking 1.7 and 8.7 minutes. Visual inspection showed an exchange of measurement error between sitting/standing and walking, as the measurement error increased simultaneously. Throughout the experimental setup, participants were instructed: lying 9 minutes (dashed reference line), sitting/standing 12.0 minutes (solid reference line), and walking 9.0 minutes (dashed reference line). The HealthPatch recorded lying for a mean of 9.2 minutes, sitting/standing 14.4 minutes, and walking 6.4 minutes (Table 1). The recorded time sitting/standing was significantly overestimated by the HealthPatch compared to the reference value, while the recorded time walking was significantly underestimated.

**Table 1:** Difference between the mean HealthPatch outcomes (device under investigation) and video data (golden standard) in the experimental setup, analyzed with the one sample t-test.

Level of mobility	HealthPatch (mean, SD)	Video data (golden standard)	p-value	95% confidence interval (lower–upper bound)
*Experimental setup*	*Minutes*	*Minutes*		
Lying	9.2 (1.1)	9.0	0.303	-0.2–0.6
Sitting/standing	14.4 (2.7)	12.0	<0.001	1.3–3.4
Walking	6.4 (2.3)	9.0	<0.001	-3.5 –-1.7

### Concurrent validity–hospital setup

**Fig 3:**
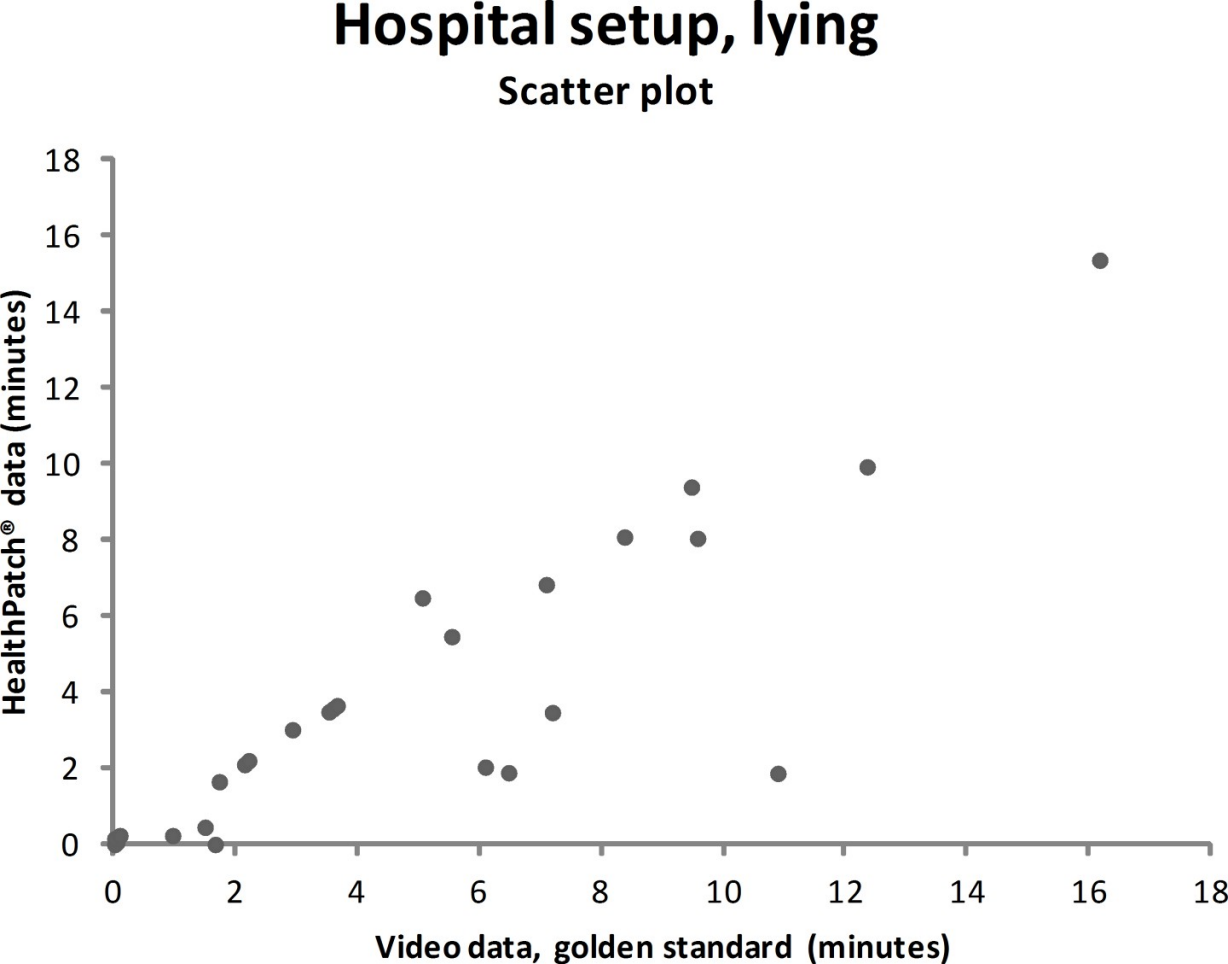
Scatter plot showing the correlation between the video data (golden standard) and HealthPatch data (device under investigation) for lying in the hospital setup (ICC agreement: 0.824).

**Fig 4:**
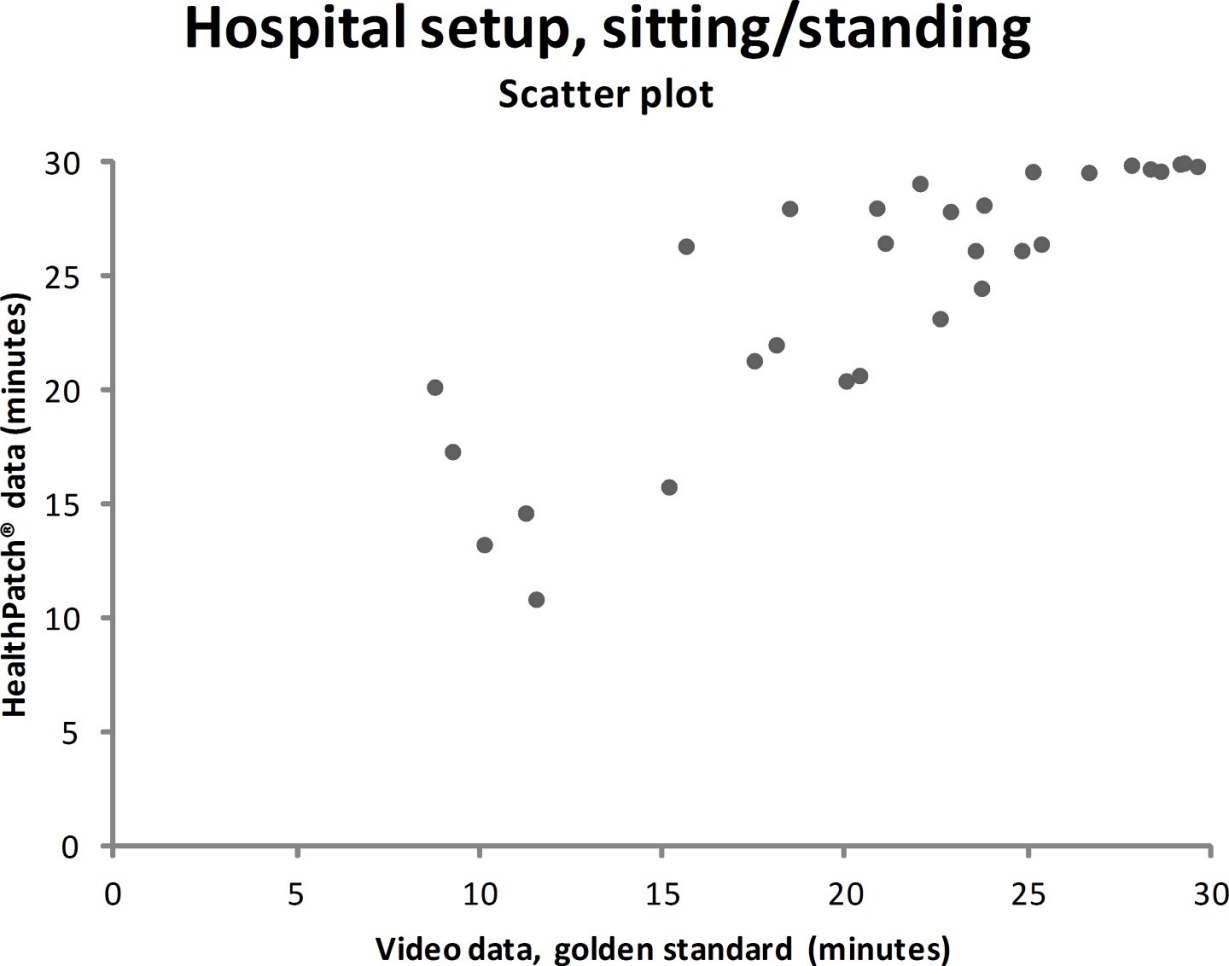
Scatter plot showing the correlation between the video data (golden standard) and HealthPatch data (device under investigation) for sitting/standing in the hospital setup (ICC agreement: 0.715).

**Fig 5:**
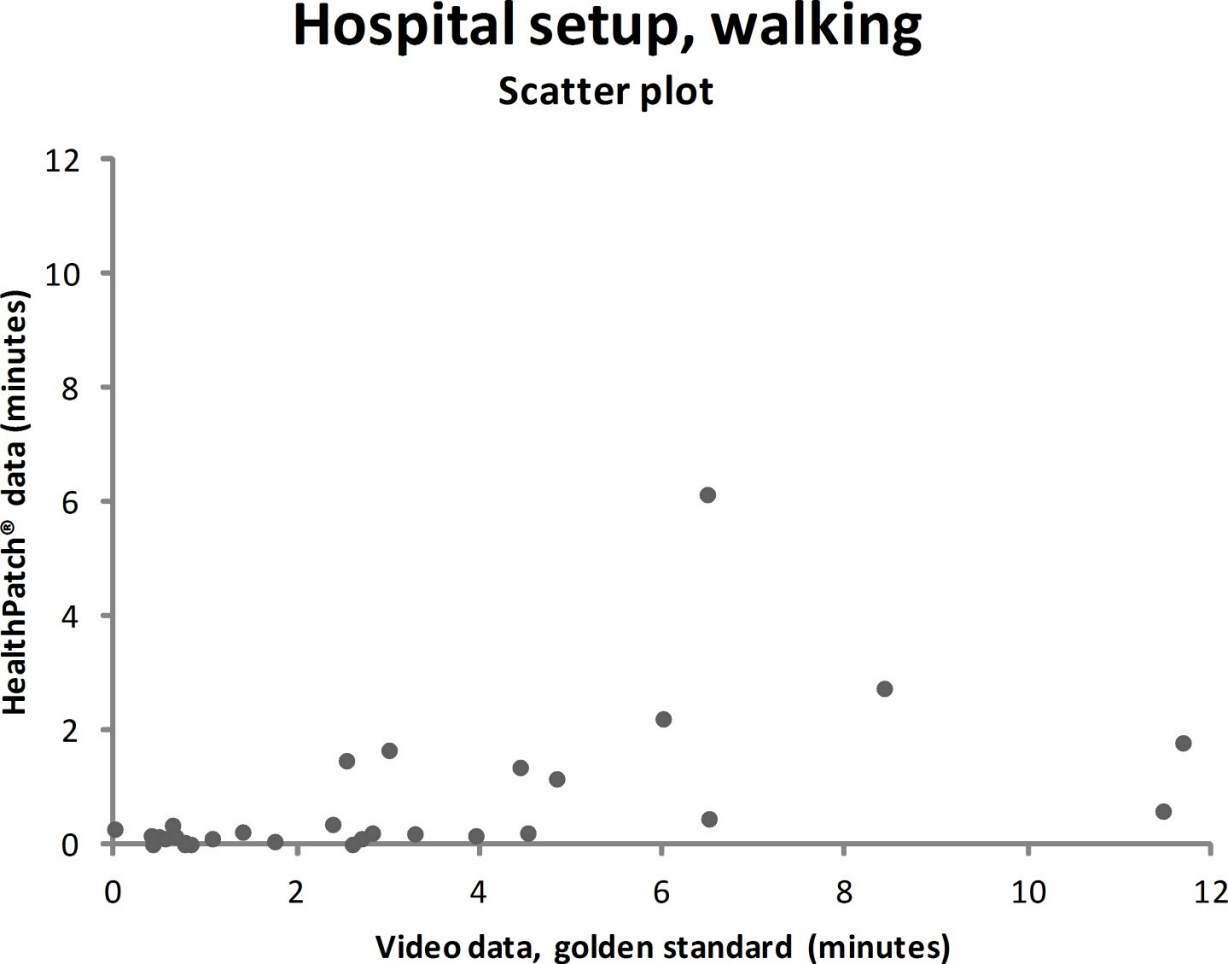
Scatter plot showing the correlation between the video data (golden standard) and HealthPatch data (device under investigation) for walking in the hospital setup (ICC agreement: 0.406).

**Fig 6:**
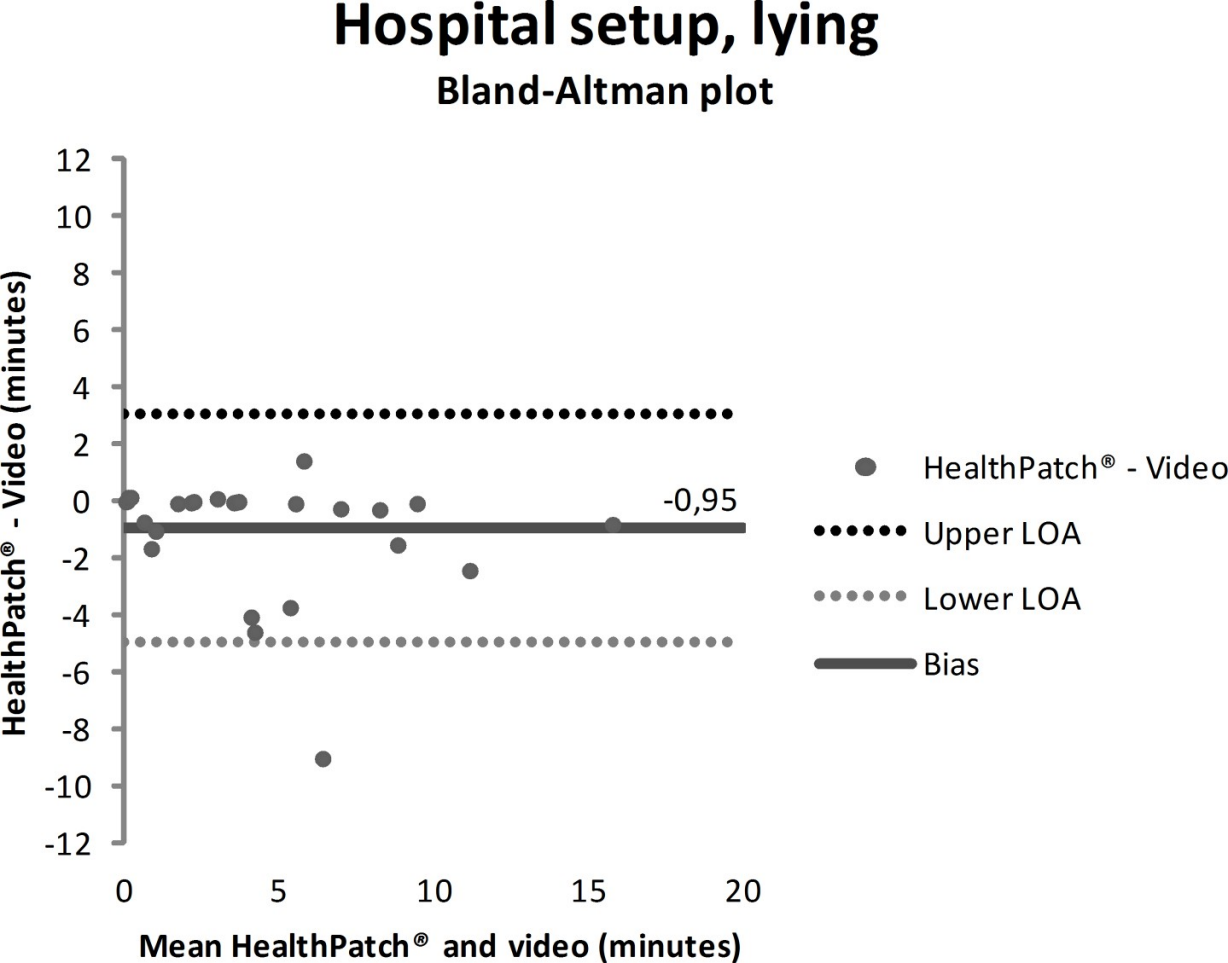
Bland-Altman plot showing mean difference (solid line) and 95% limits of agreement (dashed lines) between HealthPatch data and video data of the observed time lying in the hospital setup.

**Fig 7:**
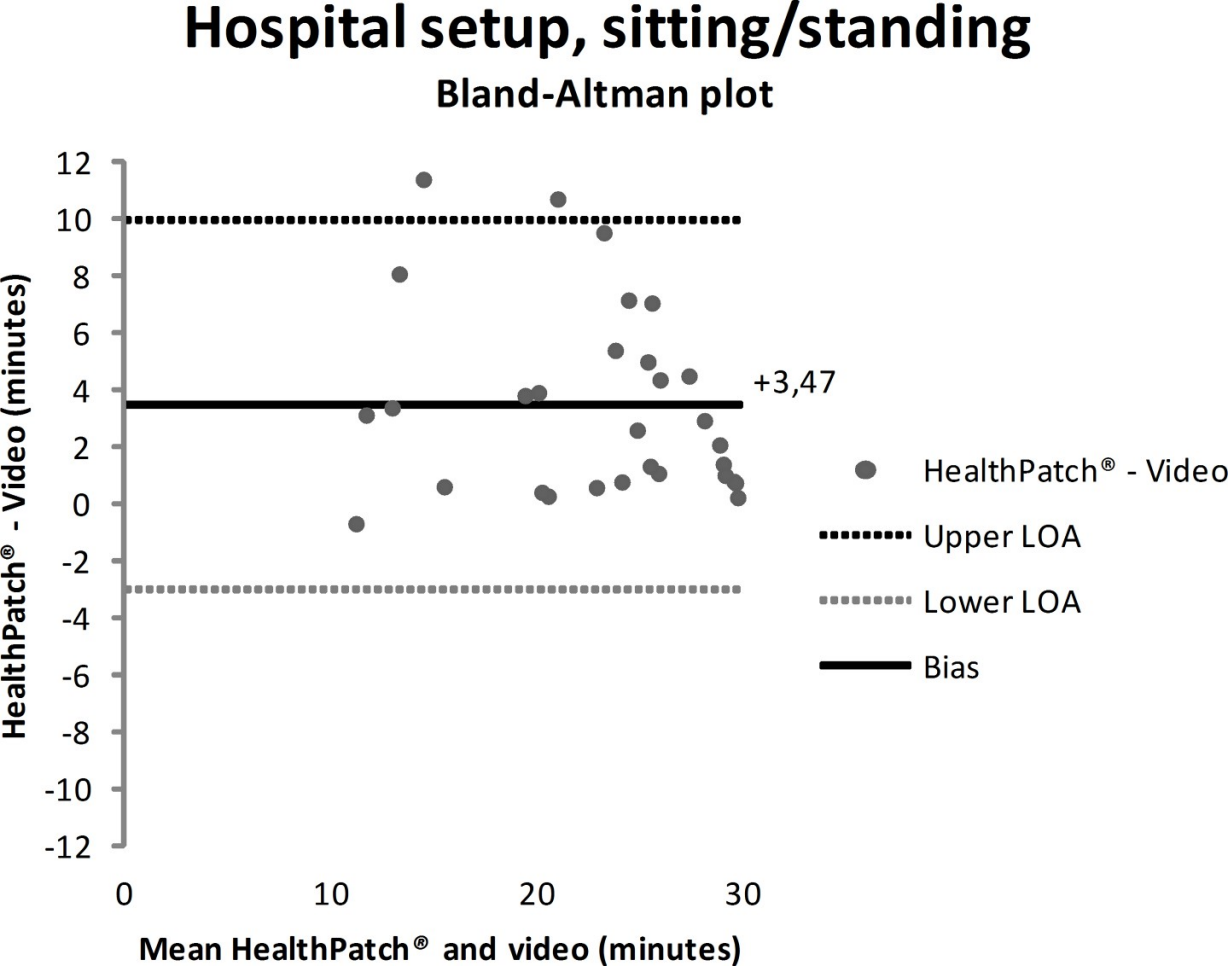
Bland-Altman plot showing mean difference (solid line) and 95% limits of agreement (dashed lines) between HealthPatch data and video data of the observed time sitting/standing in the hospital setup.

**Fig 8:**
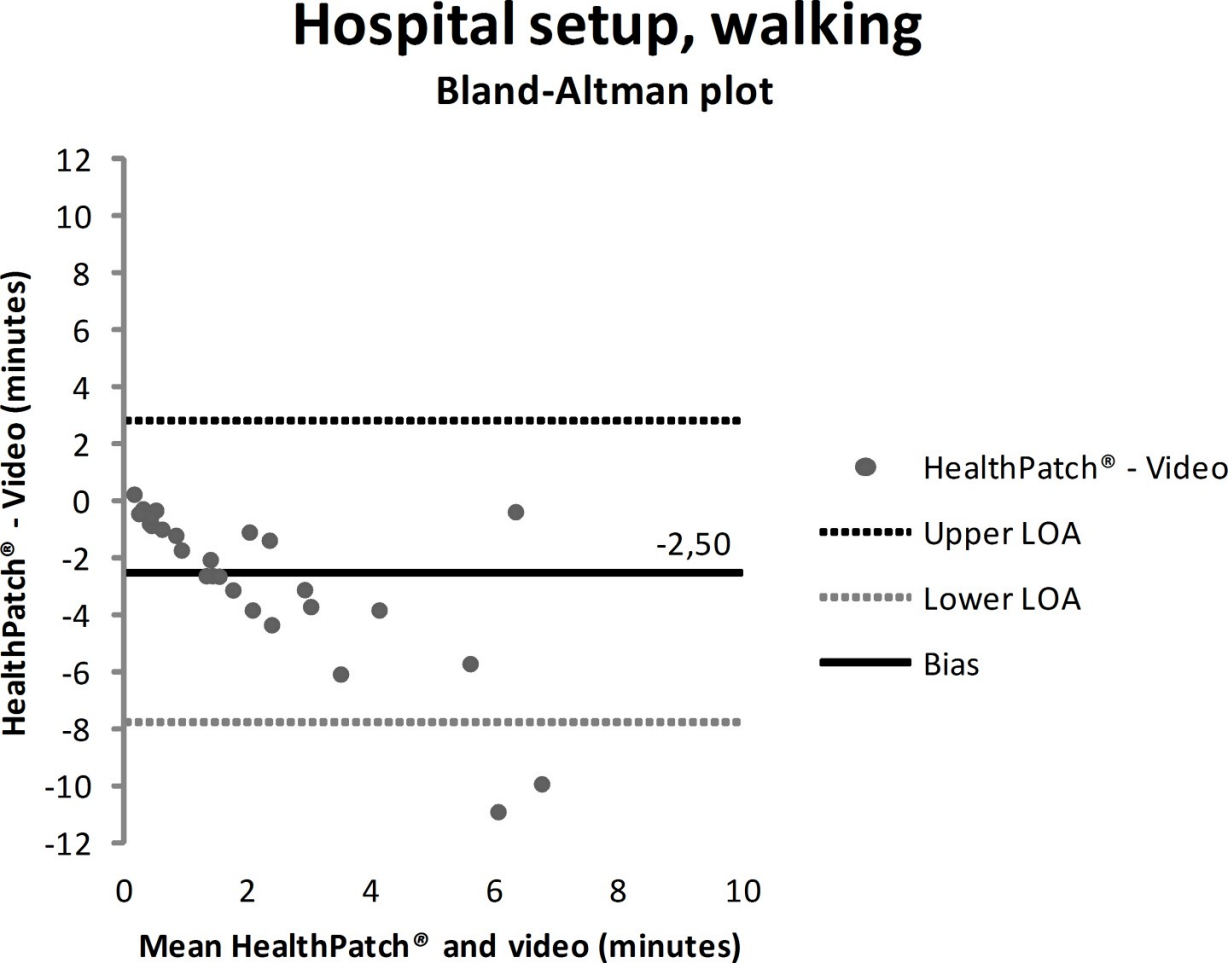
Bland-Altman plot showing mean difference (solid line) and 95% limits of agreement (dashed lines) between HealthPatch data and video data of the observed time walking in the hospital setup.

Video data during the hospital setup measurements showed that the participants were lying mean 4.7 minutes (SD: 9.0), sitting/standing mean 22.0 minutes (SD: 9.9), and walking mean 3.3 minutes (SD: 5.6). Figs 3–5 show outcomes of participants in the hospital setup at individual level. ICC’s agreement between the HealthPatch outcomes and video data were respectively 0.824 (good), 0.715 (good), and 0.406 (poor) for lying, sitting/standing, and walking (Table 2). Furthermore, Bland-Altman analysis of the HealthPatch data compared to the video data showed a mean difference of -0.95 (LOA: -4.95–3.05) when lying (in minutes), which indicated a mean underestimation of 20% of total time lying (Fig 6). Furthermore, analysis showed an overestimation of 16% of total time sitting/standing with a mean difference of 3.47 (LOA: -3.01–9.96) (Fig 7). In addition, a mean difference of -2.50 (-7.82–2.81) was observed when walking, which indicated a mean underestimation of 76% of total time walking (Fig 8). Ultimately, Bland-Altman analysis seemed to show an increase in measurement error of the HealthPatch walking data concurrent with an increase of actual time walking.

**Table 2:** Correlation between the HealthPatch data (device under investigation) and video data (golden standard) in the hospital setup, analyzed with the intraclass correlation coefficient (model 3.1 agreement).

Level of mobility	HealthPatch (mean, SD)	Video data (mean, SD)	ICC agreement	95% confidence interval (lower–upper bound)
*Hospital setup*	*Minutes*	*Minutes*		
Lying	3.7 (7.7)	4.7 (9.0)	0.824	0.779–0.860
Sitting/standing	25.5 (8.2)	22.0 (9.9)	0.715	0.486–0.827
Walking	0.8 (2.6)	3.3 (5.6)	0.406	0.182–0.567

### Comparison experimental and hospital setup

The posture detection accuracies of the HealthPatch ranged between 72.3% and 99.3% (Table 3). The most accurate posture detection was shown in the experimental setup for lying, the lowest for walking in the experimental setup. The posture was observed accurately each 30.0 minutes for a mean time of 26.2 minutes in the hospital setup and 24.4 minutes in the experimental setup. Overall, the posture detection accuracies were statistically significantly different between the experimental and the hospital setup measurements at all levels of mobility. In specific, posture detection accuracies were higher in the experimental setup except for walking.

**Table 3:** Mean differences in posture detection accuracies (in percentage) of HealthPatch data between the experimental and the hospital setup.

Posture	Experimental setup (mean, SD)	Hospital setup (mean, SD)	p-value	95% confidence interval (lower–upper bound)
Lying	99.3% (0.6)	94.0% (10.0)	0.011	-9.3%–-1.3%
Sitting/standing	98.6% (4.8)	85.4% (13.6)	<0.001	-18.8%–-7.6%
Walking	72.3% (24.9)	89.9% (9.7)	<0.001	7.7%– 27.6%

Group differences were tested with the independent samples t-test.

## Discussion

This is the first study to assess the concurrent validity of the HealthPatch to determine time lying, sitting/standing, and walking in both an experimental and a hospital setup. Experimental setup outcomes show poor validity of the HealthPatch to determine time sitting/standing and walking, indicated by statistically significant differences with reference values. The HealthPatch is able to validly monitor lying and sitting/standing in a real-world hospital setup reflected by good ICC’s. These findings implicate that the HealthPatch might be a promising tool to assess the time lying and sitting/standing of patients during hospital stay. However, the ICC between HealthPatch and video data for walking in the hospital setup is poor and Bland-Altman analysis shows a high and increasing measurement error. The posture detection accuracies show low walking detection accuracies and statistically significant differences on all posture detection accuracies between the experimental and the hospital setup. With this knowledge, the use of HealthPatch walking data could not be used interchangeable with the golden standard (video data) and should be considered as biased [24]. The statistically significant and clinically relevant underestimation of time lying by the HealthPatch could result in incorrect recommendations to walk more often, which could potentially harm patients during their hospital stay. For example, when a patient walked 33 minutes per day hospital stay, HealthPatch monitoring might suggest that the patient walked 8 minutes. In addition, the partially loss of mobility data in 7 out of 31 participants suggests that the technology readiness level of the HealthPatch is insufficient for implementation in daily practice.

Earlier research on posture detection accuracies of the HealthPatch by Chan et al. [15] shows results between 88.1% and 95.4% dependent on patch location. However, this study has not followed current methodological recommendations as described by the COSMIN group [19] and performed measurements in only one experimental setup. Pedersen et al. [11] report posture detection accuracies of two wireless monitors (thigh and ankle) in a hospital setup of 90.8%-100% when lying, 73.1%-98.6% when sitting/standing, and 96.5% when walking. Validation of those two wireless monitors is promising, though validation was not the primary aim of the study and therefore performed in a very small sample (n = 6) on limited data (lying 42 minutes, sitting/standing 30 minutes, walking 18 minutes). In addition, Baldwin et al. [26] assessed validity outcomes of the activPAL in patients after intensive care unit discharge. They present similar results to the current study as Bland-Altman analysis reflects an overestimation of the time standing and underestimation of walking. At last, the validation study of Brown et al. [21] included patients admitted at medical wards and shows high correlations of wireless monitors data with behavioural observations in lying (0.98, Pearson correlation coefficient), in sitting (0.97) and standing/walking (0.91). Despite the limitations of Pearson correlation coefficient analysis to detect systematic bias [24], these results show good potential especially for time standing/walking of participants.

### Strengths and limitations

Strengths of the current study include the analysis of video recorded data as a golden standard for mobility observation [21]. In addition, the use of a real-world hospital setup showed important differences of HealthPatch outcomes between the experimental and the hospital setup. A limitation was the inclusion of healthy participants instead of hospitalized patients which decreases the real-world generalizability. Observations in patients during hospitalization were considered too intrusive and benefits for participation were considered too low for patients as vulnerable research participants according to the declaration of Helsinki [27]. A second limitation is the use of different observers for the video data. The influence of different observers on outcomes was addressed with rigorous training, discussion of posture definitions, and standardized reporting. Furthermore, the inter-observer reliability analysis shows excellent agreement (ICC: 0.971–0.999). At last, the loss of data in one participant might have been prevented by shaving his chest hair as recommended by the manufacturer.

### Recommendations for future research

Firstly, the differences between outcomes in the experimental and real-world hospital setup show the need for a real-world setup to determine the validity of accelerometry. The validity for examination of time lying, sitting/standing, and walking is different between setups indicated by analysis on both individual (Figs 3–5 versus Figs 6–8) and group level (Table 3). In addition, a secondary analysis of the video data showed for example that the low posture detection of walking in the experimental setup (72.3%) compared to the hospital setup (89.9%) could be the result of overly cautious and controlled transfer from sitting to walking by participants 2, 27, and 30 in the experimental setup. Therefore, future research on validity of accelerometry for hospital use should be performed in a real-world hospital setup [14]. Secondly, knowledge of reliability outcomes is needed to be able to early discriminate patients at (risk for) low mobility and provide mobility stimulating interventions if possible.

## Conclusions

Assessment of mobility in patients during hospital stay is of great clinical importance. Valid and accurate assessment of in-hospital mobility contributes to early detection of patients at risk for development of new disability during their hospital stay as a result of physical inactivity. Furthermore, mobility assessment is crucial for monitoring purposes in interventions aiming to increase in-hospital mobility of patients. Wireless devices with accelerometry, such as the HealthPatch, provide an opportunity to continuously monitor mobility in patients without mobility limitations.

This is the first study to assess the concurrent validity of an accelerometer to monitor levels of mobility in both an experimental and a hospital setup. Overall, the results show a good validity of the HealthPatch to monitor lying and poor validity for sitting/standing and walking. In addition, the validity outcomes were less favourable in the hospital setup. The use of an experimental and a hospital setup within this study provides a blue-print for future studies to analyze validity of accelerometry.

## Supporting information

S1 Fig Detailed map of the research area, a real-world hospital room. The measurements were performed in the research zone with a hospital bed, nightstand, table, two seats, and a closet. Two cameras (GoPro HERO 4) were used to videotape the mobility of participants.
(TIF)

S1 Dataset Raw data case report forms. (ZIP)

S2 Dataset Concurrent validity–experimental setup data. (ZIP)

S3 Dataset Concurrent validity–hospital setup data. (ZIP)

S4 Dataset Comparison experimental and hospital setup data. (ZIP)
