# Supplementary figures and images for: Validation of a wireless patch sensor to monitor mobility tested in both an experimental and a hospital setup: A cross-sectional study
Source: PLoS One. 2018 Oct 25;13(10):e0206304. doi: 10.1371/journal.pone.0206304 (PMC6201929; doi:10.1371/journal.pone.0206304)

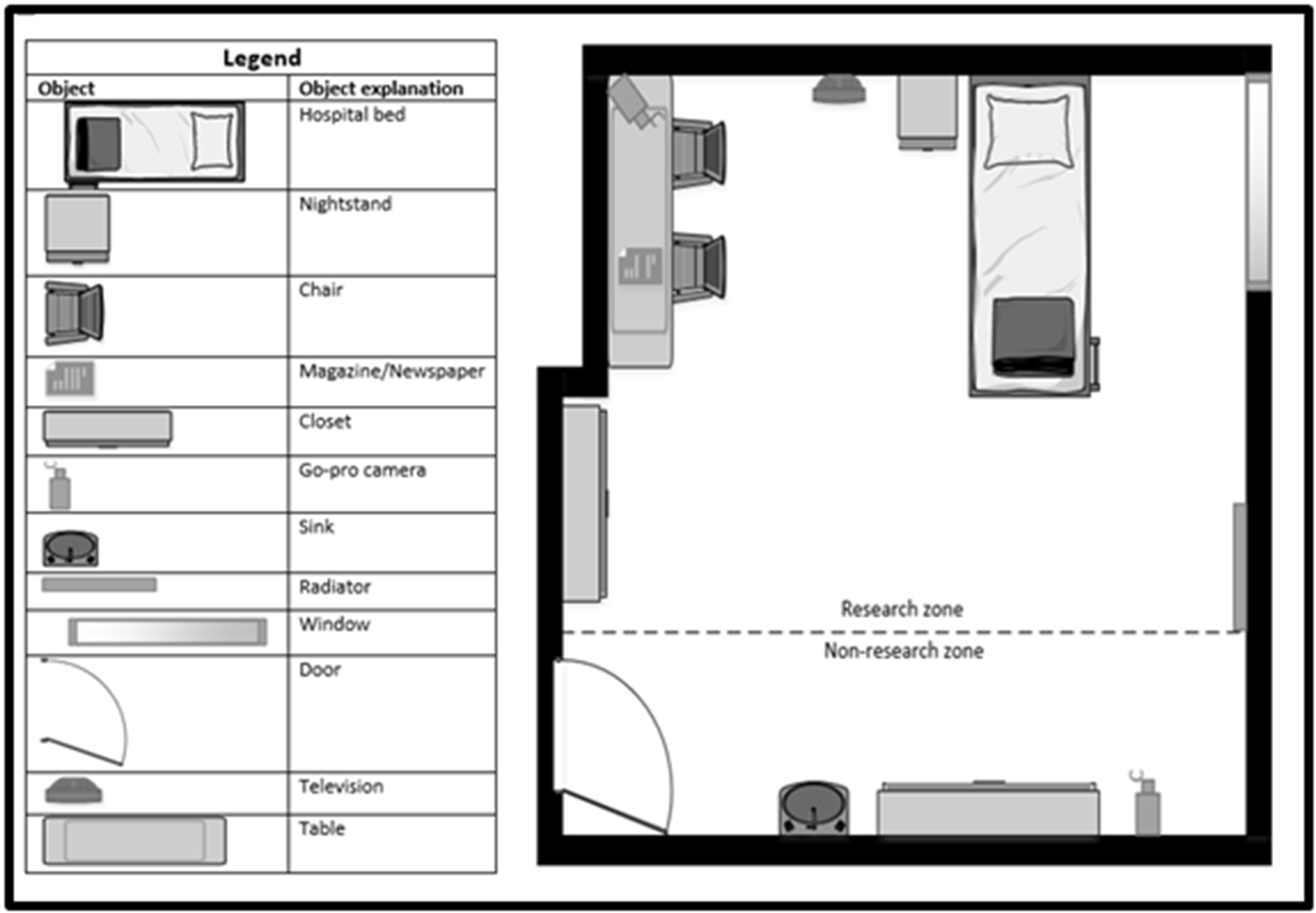

Supplement: S1 Fig — The measurements were performed in the research zone with a hospital bed, nightstand, table, two seats, and a closet. Two cameras (GoPro HERO 4) were used to videotape the mobility of participants. (TIF) [file pone.0206304.s001.tif]
